# The complete mitochondrial genome of *Glycyrrhiza uralensis* Fisch. (Fabales, Leguminosae)

**DOI:** 10.1080/23802359.2021.1872432

**Published:** 2021-02-09

**Authors:** Yan-Yun Yang, Sheng-Nan Li, Liang Xu, Yan-Ping Xing, Rong Zhao, Gui-Hua Bao, Ting-Ting Zhang, Da-Chuan Zhang, Yue-Yue Song, Wu-Liji Ao, Ting-Guo Kang

**Affiliations:** a School of Pharmacy, Liaoning University of Traditional Chinese Medicine, Dalian, China; b School of Mongol Medicine, Inner Mongolia University for Nationalities, Tongliao, China

**Keywords:** Mitochondrial genome, *Glycyrrhiza uralensis*, Leguminosae

## Abstract

The complete mitochondrial genome of an important medicinal plant *Glycyrrhiza uralensis* Fisch. is reported for the first time. The mitochondrial genome sequence of *G. uralensis* was 463,869 bp in length and had a GC content of 45.19%. The genome contained 40 protein-coding genes (PCGs), 30 transfer RNAs (tRNAs), and three ribosomal RNAs (rRNAs). The phylogenetic tree was built based on 25 plants, using the maximum-likelihood method. These data will provide certain help to determine the taxonomic status of *G. uralensis*.

*Glycyrrhiza uralensis* Fisch. is a perennial herb belonging to family Leguminosae, widely distributed from Central Asia to Northeast China. *G. uralensis* was first published in ‘Shen Nong's Materia Medica’, which has a history of 2000 years. It is known as the ‘King of Traditional Chinese Medicine’ in China. Studies on the effective biological activity of *G. uralensis* include antitumor (Aipire et al. 2017), antiviral (Wang et al. 2013), anti-inflammatory (Yang et al. 2017), and antibacterial (Chen et al. 2019), etc. Among the numerous researches on *G. uralensis*, most of them have focused on its pharmacological analysis and component comparative analysis. At present, whole-genome sequencing and discussion of some key genes have also been carried out (Liu et al. 2015; Mochida et al. 2017).

A sample was collected from fresh leaves of *G. uralensis* in Dalian, China (E 121°87′74.06″, N 39°06′18.21″). The *G. uralensis* plant samples and genomic DNA were stored in the herbarium of Liaoning University of Traditional Chinese Medicine (*G. uralensis* number: 10162200520025LY). *G. uralensis* genome was sequenced using a combination of the Nanopore platform (PromethION, Oxford Nanopore Technologies, Oxford, UK) and Illumina NovaSeq platform (Illumina, San Diego, CA). First, we used ABySS v2.0.2 (Simpson et al. 2009) to perform genome assembly with multiple-Kmer parameters and received the optimal results of the assembly. Second, BLASR (Chaisson and Tesler 2012) was used to map the preliminary assembly results to the Nanopore long reads. Then, SPAdes v3.10.1 (Bankevich et al. 2012) was used to assemble them together to construct contigs (Scaffolds), followed by error correction using Pilon v1.21 (Walker et al. 2014). The Nanopore assembled sequences were then checked if the sequence has overlapped and connected between them. The mitochondrial genome information of *G. uralensis* will provide data for further analysis of evolutionary history.

The mitochondrial genome of *G. uralensis* was sequenced by using the Illumina platform and Nanopore platform, which generated 26.5 million reads and 4.0 Gb raw data, respectively. Finally, the complete mitochondrial genome of *G. uralensis* was a circular form of 463,869 bp with an average read coverage of 3753× (coverage of Illumina reads was 3641.6×, coverage of Nanopore reads was 112.2×), and had a GC content of 45.19%. The mitochondrial genome encoded 73 unique genes, including 40 protein coding genes and 33 protein non-coding genes. The total length of the protein coding genes was 34,074 bp, accounting for 7.35% of the total length of the genome. The total length of non-coding proteins was 5129 bp (1.61%), including 30 transfer RNA (tRNA) genes and three ribosomal RNA (rRNA) genes (*rrn18*, *rrn5*, and *rrn26*). In addition, we found eight genes with introns (*ccmFc*, *nad5*, *rps3*, *rps10*, *nad1*, *nad7*, *nad2*, and *nad4*) containing 22 introns in total.

In order to understand the phylogenetic location of *G. uralensis*, we downloaded the mitochondrial genomes of 24 other plants and *G. uralensis* in NCBI to construct the phylogenetic tree with *Ginkgo biloba* as the outgroup. We use the MUMmer (Marcais et al. 2018) and BLAT software (Kent 2002) to do global alignment and local alignment between sample sequence and the reference genome under default parameters, and then manually optimized. The maximum-likelihood (ML) methods were performed for the genome-wide phylogenetic analyses using PhyML v3.0 (Guindon et al. 2010). Nucleotide substitution model selection was estimated with jModelTest v2.1.10 (Darriba et al. 2012) and Smart Model Selection in PhyML. The model GTR + I+G was selected for ML analyses with 1000 bootstrap (BS) replicates to calculate the BS values of the topology. The results tree was treated with iTOL v3.4.3 (Letunic and Bork 2016). It was found that *G. uralensis* and *Medicago truncatula* were closely clustered together (Figure 1). At present, the available mitochondrial genome of angiosperm is still limited, which hinders our comprehensive understanding of the evolution of angiosperm mitochondrial genome. Besides the sequence level, plant mitochondrial genome can provide phylogenetic information at the structural level. These data will provide a basis for us to analyze the phylogenetic position of *G. uralensis*.

**Figure 1.**
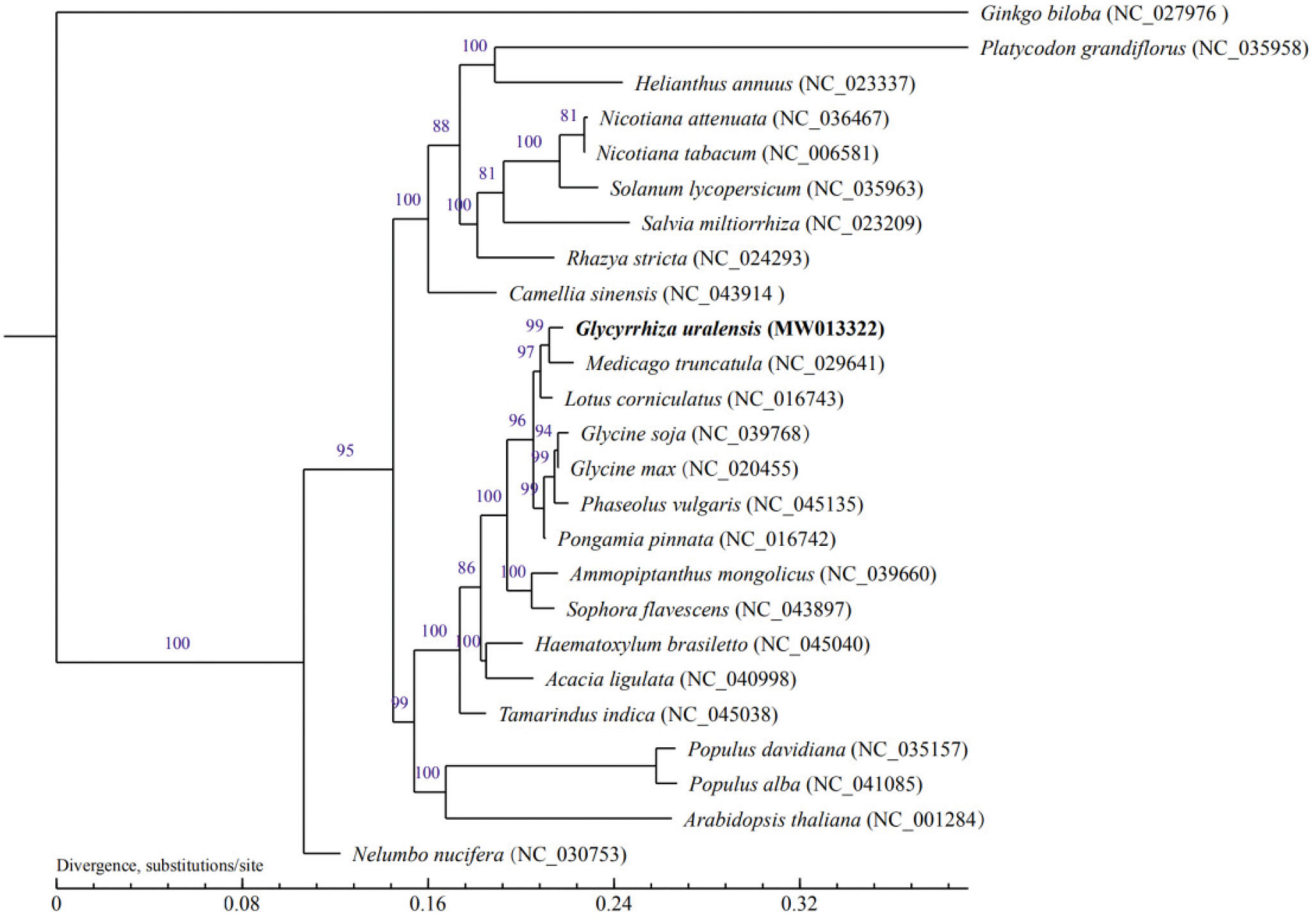
Maximum-likelihood (ML) tree based on the mitogenome sequence of *Glycyrrhiza uralensis* (MW013322) with 24 other species, the bootstrap supports are shown on each node.

## Data Availability

The genome sequence data that support the findings of this study are openly available in GenBank of NCBI at https://www.ncbi.nlm.nih.gov/ under the accession no. MW013322. The associated BioProject, SRA, and Bio-Sample numbers are PRJNA679165, SRX9530379, and SRS7737028, respectively.
